# Supplementary material for: Galangin Rescues Alzheimer’s Amyloid-β Induced Mitophagy and Brain Organoid Growth Impairment
Source: Int J Mol Sci. 2023 Feb 8;24(4):3398. doi: 10.3390/ijms24043398 (PMC9960784; doi:10.3390/ijms24043398)
Supplement: Supplementary file 1 [file ijms-24-03398-s001.zip › ijms-2177739-supplementary.pdf]

International Journal of *Molecular Sciences*

MDPI

Article

Alzheimer's Amyloid-β impairs mitophagy and growth of brain organoids which could be rescued by dietary flavonoid galangin

Ru Zhang[1], Juan Lu[1], Gang Pei[1,2,3], Shichao Huang[1,*]

[1] State Key Laboratory of Cell Biology, Shanghai Institute of Biochemistry and Cell Biology, Center for Excellence in Molecular Cell Science, Chinese Academy of Sciences, Shanghai 200031, China
[2] Shanghai Key Laboratory of Signaling and Disease Research, Laboratory of Receptor-Based Biomedicine, The Collaborative Innovation Center for Brain Science, School of Life Sciences and Technology, Tongji University, Shanghai 200070, China
[3] Institute for Stem Cell and Regeneration, Chinese Academy of Sciences, Beijing 100045, China
* Correspondence: huangshichao@sibcb.ac.cn (S.H.)

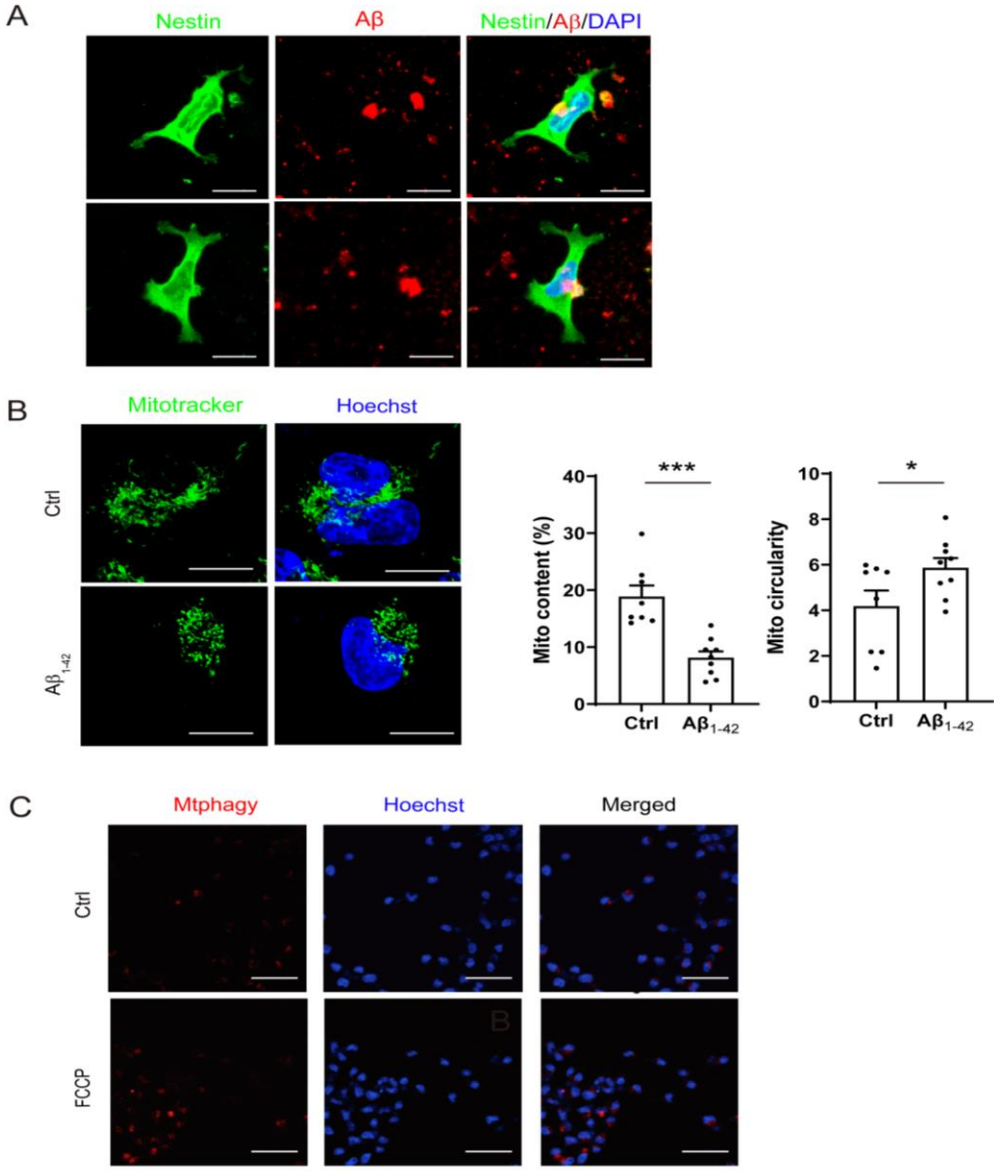

Figure S1. (A) Immunofluorescence staining of Aβ and Nestin on Aβ-treated NPCs. Scale bar, 20 μm. (B) Images and quantifications of MitoTracker staining on Aβ-treated NPCs. Scale bar, 10 μm. (C) Mitophagy staining of mitophagy induction (FCCP). Scale bar, 50 μm. * $p < 0.05$, *** $p < 0.001$
